# Characterization of Volume-Based Changes in Cortical Auditory Evoked Potentials and Prepulse Inhibition

**DOI:** 10.1038/s41598-017-11191-3

**Published:** 2017-09-11

**Authors:** Thomas Potter, Sheng Li, Thinh Nguyen, Trac Nguyen, Nuri Ince, Yingchun Zhang

**Affiliations:** 1 Department of Biomedical Engineering, University of Houston, Houston, TX USA; 2 Department of Physical Medicine and Rehabilitation, University of Texas Health Science Center – Houston, McGovern Medical School, and TIRR Memorial Hermann Research Center, Houston, TX USA

## Abstract

The auditory evoked startle reflex is a conserved response resulting in neurological and motor activity. The presence of a mild prepulse immediately before the main pulse inhibits startle responses, though the mechanism for this remains unknown. In this study, the electroencephalography (EEG) data recorded from 15 subjects was analyzed to study the N1 and P2 components of cortical auditory evoked potentials (CAEPs) evoked by 70, 80, 90, 100, and 110 dB stimuli both in the presence and absence of 70 dB prepulses. Results without a prepulse showed an evolution of N1 amplitudes, increasing with stimulus intensity and showing largely significant differences. Results from prepulse trials only showed noteworthy changes in peak-to-peak amplitude in the 100 dB condition. Prepulse and non-prepulse conditions were then compared using peak amplitudes and theta power. Prepulse conditions significantly decreased the amplitude for both components in the 110 dB condition, i.e., pre-pulse inhibition, but significantly increased the N1 amplitude in the 70 dB condition, i.e., pre-pulse facilitation. Similarly theta band power significantly increased in the 70 dB prepulse condition and significantly decreased in the 110 dB prepulse condition. These results expand the basis of knowledge regarding how CAEPs change and elaborate on their neural function and representation.

## Introduction

Audition is a complex process through which sounds are transduced to electrical signals in the brain, proceeding to affect neural activity and behavioral responses. Beyond straightforward mechanisms, the sense of hearing also has a broad impact on the psychological function and cognition^1–3^, making the study of objective audition markers essential. The Cortical Auditory Evoked Potential (CAEP), one such marker, is a long-latency potential that is generated in response to auditory stimuli. Believed to originate from the primary auditory cortex, this event-related potential is readily observable from scalp EEG in frontocentral locations^4^. While CAEPs are generally regular and consist of multiple stereotyped components, their exact presentation can vary depending on the chosen stimulus^5, 6^. The three most prominently observed CAEP components are the P1, N1, and P2 peaks, which can be observed at 50, 100, and 150 ms after stimulus onset, respectively^4^. This long latency, paired with their ease of detection, has led to the use of CAEPs as objective markers for hearing, cognition, and a variety of both physical and mental illnesses.

As a direct marker of auditory function and cognition, CAEPs have been largely utilized to assess aural function. When applied to the direct detection of auditory stimuli, CAEPs were found to be indicative of sound discrimination capabilities as measured by just noticeable differences and mismatch responses^7^. Further tests have shown that both the amplitude and latency of the P1 CAEP component were correlated with speech perception in adults and children^5, 8–10^. Beyond the examination of functional hearing in healthy subjects, CAEPs have also been used to assess hearing loss^11^ and the function of cochlear implants and hearing aids. Recent research suggests that CAEPs may further serve as objective tools to aid in the assessment and programming of Cochlear implants^12^, with later experiments revealing correlations between CAEP scores and Mandarin Early Speech Perception tests in children after cochlear implantation surgery^13^. Similarly, experiments have shown increased CAEP presence in subjects with bilateral hearing loss after the hearing aids were applied, particularly with reference to /g/ and /t/ sounds^14^. Clinical application for the cortical auditory evoked potential can be even further extended to the assessment of the ill^15^, as P2 amplitude has shown potential as an indicator of early-stage cognitive impairment, while mismatch negativity within the CAEP may be a good indicator of the likelihood of recovery in comatose patients^16, 17^. Despite their clinical relevance and the ease of collection, CAEPs are not well characterized – though their overall shape is stereotyped, how the potential changes in response to differing stimuli has yet to be fully understood.

Experiments focusing on cortical auditory evoked potentials have used a variety of stimulus intensities in their tests. An underlying assumption, then, is that CAEPs are relatively stable across volume levels. Unfortunately, the process of audition is known to be somewhat volatile, as seen in phenomena such as the auditory startle reflex (ASR), which has become a research focus in recent years^18, 19^. The auditory startle reflex induces a rapid, involuntary disruption to physical activity and cognitive processing, marked by muscle spasm and subsequent increases in the subject’s heart rate and skin conductance^18^. Muscle activity in these cases includes large-scale contractions throughout the body, though the extent to which this occurs may be directly linked to stimulus intensity. Unlike the CAEP, which originates from the primary auditory cortex, startle responses are believed to be generated within a region of the caudal pons known as the nucleus reticularis pontis caudalis (nRPC)^20, 21^. As the nRPC is not linked to any specific modality, startle reflexes may be induced through auditory, somatosensory, or visual means^18, 22, 23^. Once initiated, regardless of the stimulus type, signals proceed to affect both neural activity and potentiation in the cortex and initiate reflexive motor contractions^19, 24^. While the startle reflex is well-conserved and fairly stereotypical response, alterations can be observed based on the psychological or physical state of the subject. Specifically, degrees of difference can be observed across multiple diseases and disorders, including stroke^25^, dystonia^26^, autism^27^, and Tay-Sachs disease^28^. One curious phenomenon that has been recorded across multiple studies is the alteration of the startle reflex in the presence of a milder, preceding stimulus. This may manifest in two forms – prepulse facilitation (PPF)^29^ and prepulse inhibition (PPI)^30, 31^ – which are believed to represent separate processes^29^. Prepulse facilitation has typically been observed in situations with very short^29, 32^ and very long prepulse intervals^33^, resulting in an increase to startle intensity. Prepulse inhibition on the other hand, is induced by a non-startling stimulus occurring 30–500 ms prior to the startling pulse^34^ and leads to a reduced startle response. PPI serves as a remarkably conserved and consistent neurological marker: it is exhibited by all mammals, does not show any characteristics of conditioning or habituation^35^, and is consistent across all sensory modalities^36^, even occurring when the pulse and prepulse are presented via different modes^37^. At the current time, there are two complementary hypotheses that describe how PPI functions: namely, the interruption and protection hypotheses^38, 39^. In brief, the interruption hypothesis states that startling pulses interrupt the cognitive processing of the prepulse stimulus and the protection hypothesis theorizes that PPI serves to “protect” the cognitive processing of the early stimulus by limiting the processing and reaction to the secondary startling pulse. More recent experiments have provided support for these hypotheses^40^, showing the potential for prepulse inhibition to effect auditory evoked theta oscillations^41^ and providing a clinical basis for studying PPI^42, 43^. Unfortunately, despite these advances, studies focused on PPI have not addressed how it changes across a full spectrum of auditory stimulus intensities. Most studies have instead focused a small number of stimulus amplitudes with little inter-study regularity^40, 41, 44–46^, despite the fact that PPI presentation has shown to vary with stimulus properties and experimental environment^47^. This leaves a fundamental gap when attempting to address the underlying mechanisms and representations of the PPI phenomenon.

In this paper, we seek to further elucidate the core mechanisms of the startle response and prepulse inhibition using a paradigm that compares the N1 and P2 CAEP components evoked by sounds with varying intensities in the presence and absence of a non-startling prepulse, using the theta oscillation as a secondary marker of auditory effect. Based on current knowledge regarding audition and the startle response, it is anticipated that component amplitude will vary with stimulus intensity. When prepulses are added to the signal, it is further hypothesized that CAEP component amplitudes will be reduced, especially when observed at amplitudes that typically cause a startle reflex (>95 dB). This research, ideally, will expand the basis of knowledge within these research areas, allowing us to better understand both the phenomena themselves and the factors that affect them in clinical work.

## Results

### CAEP evolution

**Figure 1:**
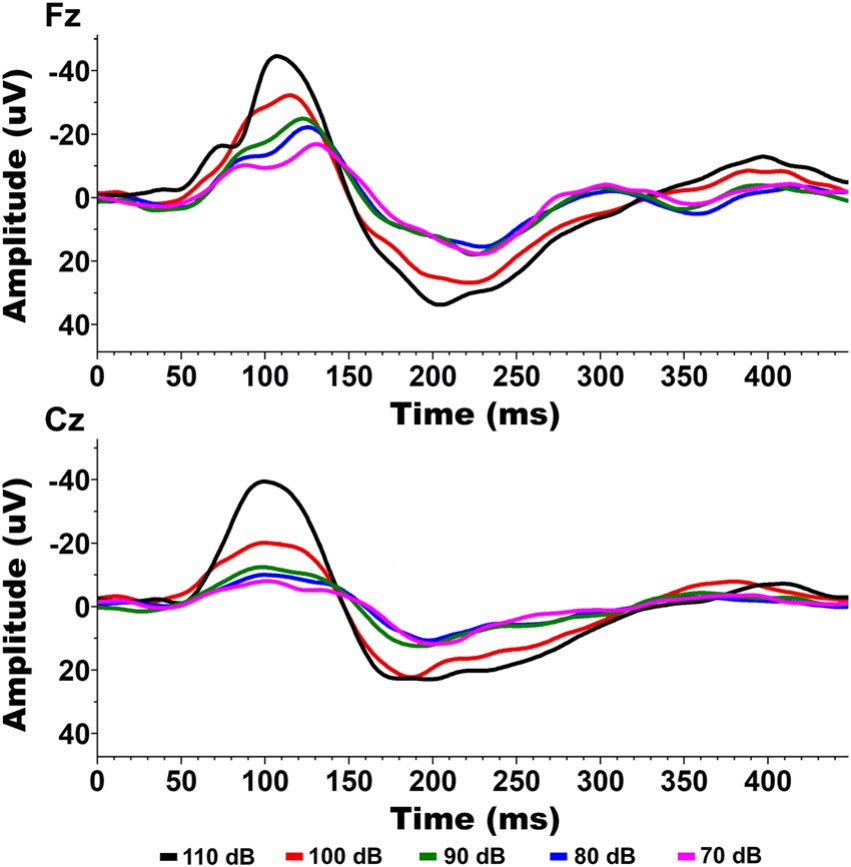
Representative waveforms from the non-prepulse condition (Subject C). A clear hierarchy is shown, as peak amplitude increases with stimulus intensity in both Fz and Cz channels.

**Figure 2:**
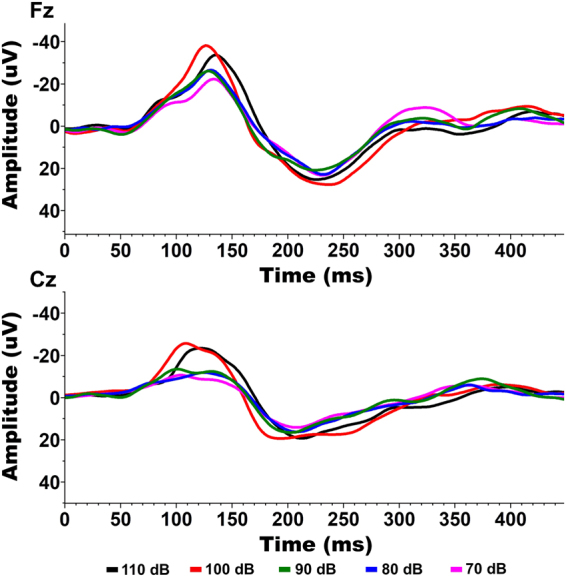
Representative waveform from the prepulse condition (Subject C). No clear hierarchy is present, with most decibel levels producing peaks of similar amplitude. The 100 dB condition remains elevated in both Fz and Cz channels.

**Figure 3:**
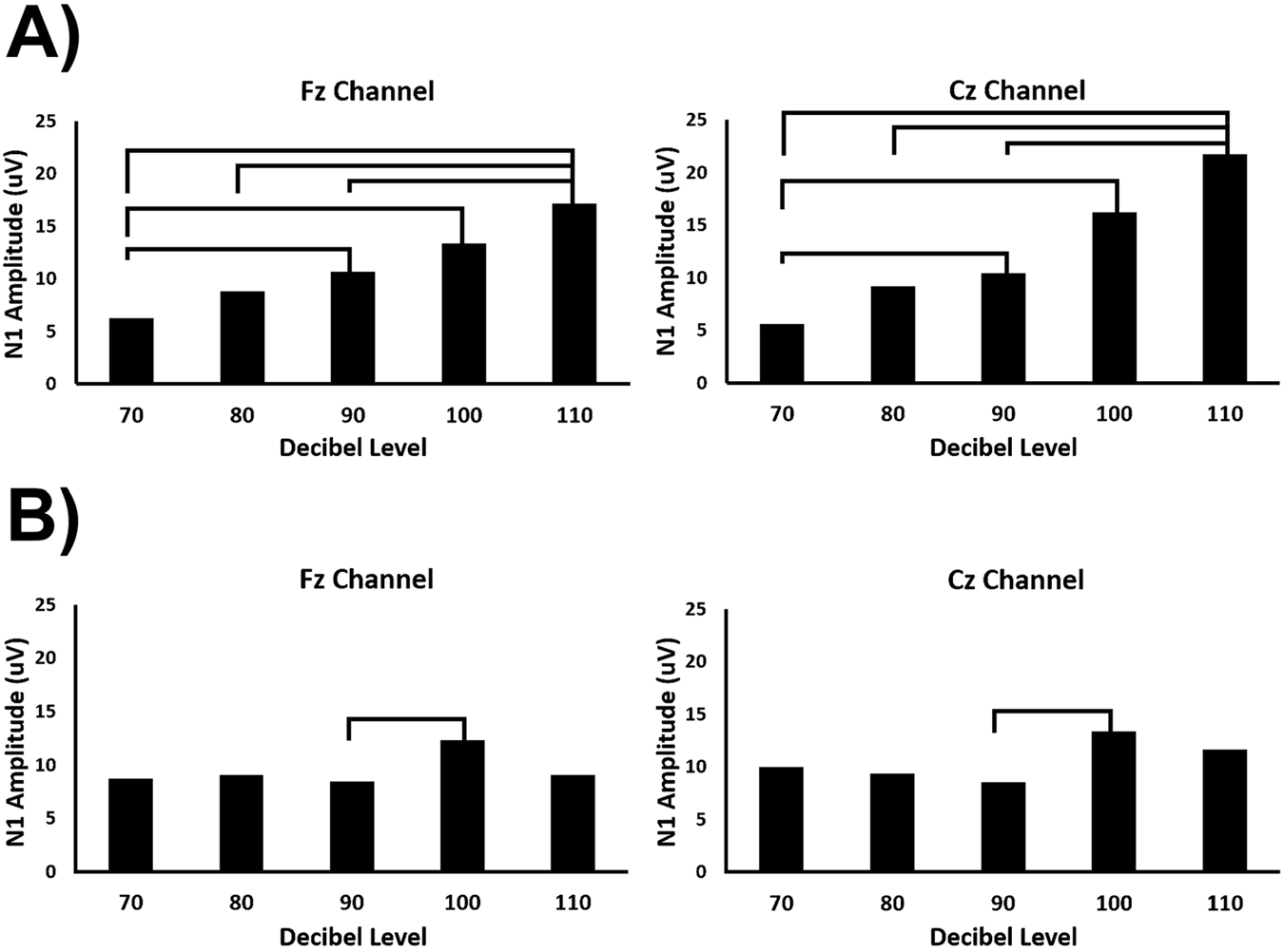
N1 amplitudes measured across decibel levels in the Fz and Cz channels without (**A**) and with (**B**) a prepulse. Significant differences between decibel levels are indicated by bars at the top

When observed qualitatively, the N1 (and to a lesser extent P2) showed a hierarchical presentation in response to increasing volume (i.e. amplitudes seemed follow a general trend that 110 dB > 100 dB > 90 dB > 80 dB > 70 dB) (Fig. 1). This trend was not as clear in the presence of a prepulse, with many decibel levels appearing similar (Fig. 2). ANOVA results for the N1 amplitude showed significant main effects for volume level changes in both the Fz (F = 3.9073, P = 0.004867) and Cz channels (F = 8.4120, P = 0.000004). Contrastingly, ANOVA results for the P2 amplitude did not show significant main effects of volume level in either the Fz or Cz channels. Theta power also did not show significant differences in relation to volume level in the Fz channel but did show a significant main effect in the Cz channel (F = 3.5238, P = 0.008978). Based on the significant main-effects, individual FDR-controlled T-tests comparing the N1 amplitudes across volume levels were performed. After correction for false discovery rate, a consistent set of comparisons showed significant statistical differences (p < 0.05) in the both the Fz and Cz Channels. These include the 70–90 (Fz: p = 0.01544, Cz: p = 0.01770), 70–100 (Fz: p = 0.00358 Cz: p = 0.002261), 70–110 (Fz: p = 0.00341, Cz: p = 0.00297), and 80–110 (Fz: p = 0.00341, Cz: p = 0.00302), and 90–110 (Fz: p = 0.00716, Cz: p = 0.00302) dB comparisons (Fig. 3A). In the presence of a prepulse, only the 90–100 dB comparison showed a significant difference, observable in both channels (Fz: p = 0.03872, Cz: p = 0.03872) (Fig. 3B).

### Prepulse vs Non-Prepulse

**Figure 4:**
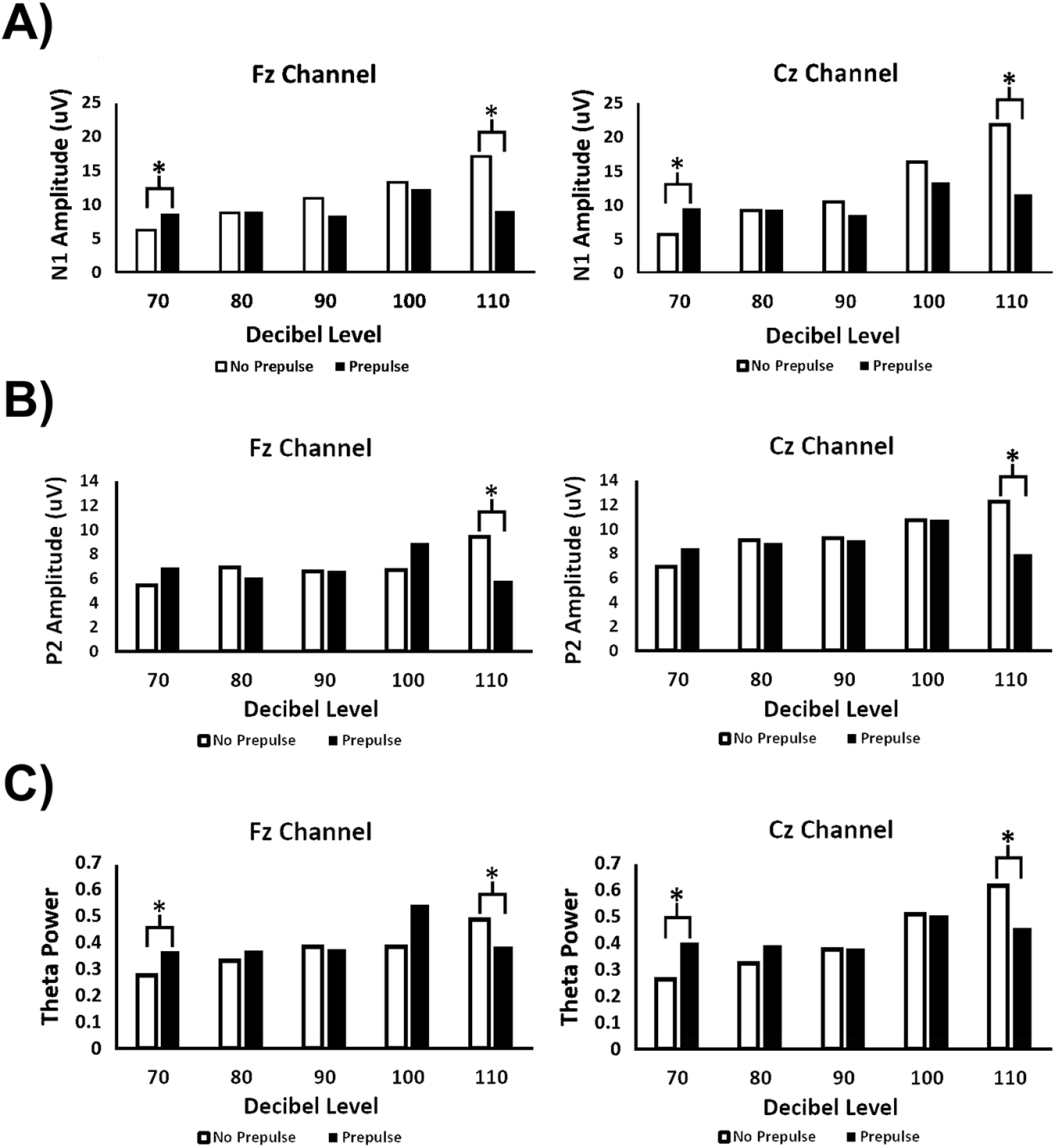
Amplitude differences between the prepulse and non-prepulse conditions for the (**A**) N1 Amplitude, (**B**) P2 Amplitude, and (**C**) Theta power. The presence of a prepulse consistently resulted in a significant decrease in amplitude and power in response to the 110 dB stimulus, while most other comparisons were non-significant. The presence of a prepulse did also show a propensity to increase amplitudes and power in response to the 70 dB stimulus, though only increases in the theta amplitude were statistically significant after correcting for multiple comparisons.

Two-way ANOVA results for the N1 amplitudes showed non-significant main effects for prepulse presence in the Fz and Cz, though significant interactions with decibel level were found in both the Fz (F = 2.4674, P = 0.04764) and Cz channels (F = 3.8978, P = 0.004942). P2 amplitudes also showed a non-significant main effect for prepulse in both the Cz and Fz channels with no significant interactions. Theta power reflected this lack of significant prepulse main effects as well, with non-significant interactions. FDR-corrected t-tests showed that the presence of a prepulse significantly decreased the N1 and P2 amplitudes at 110 dB in both the Fz (N1: p = 0.003043, P2: p = 0.017703) and Cz (N1: p = 0.02261, P2: p = 0.02261) channels (Figs 4A and 4B), with similar decreases in theta power observed at the 110 dB in both Fz (p = 0.007582) and Cz (p = 0.007582) channels (Fig. 4C). In contrast, the presence of a prepulse with the 70 dB stimulus significantly increased the N1 amplitude in the Fz and Cz channels (Fz: p = 0.04953, Cz: p = 0.02261) (Fig. 4A), along with the theta power in both Fz (p = 0.04400) and Cz (p = 0.03872) channels (Fig. 4C).

## Discussion

The phenomena of startle reflexes and prepulse inhibition are both well recognized and conserved across a variety of models and disease conditions. Despite this, many questions remain regarding the neural mechanics that underlie these responses. The experiments performed here have sought to systematically explore the evolution of cortical auditory evoked potentials (CAEP) in response to different intensities and how the addition of a mild prepulse changes these dynamics. In general, the results of this study indicate that the P2 component is more resilient to changes in decibel level or prepulse presence than the N1 or Theta oscillations – it showed no significant main effects or interactions at either channel and exhibited fewer significant differences when prepulses were introduced. The N1 and theta oscillations may, on the other hand, be linked – the patterns observed in one were generally observable in the other, with similar significant contrasts. Finally, neither the 80, 90, nor 100 dB stimuli showed any significant change with the presence of a prepulse, regardless of channel or peak, indicating that CAEPs evoked by these decibel levels may be more reliable biomarkers than those from the 70 or 110 dB stimuli.

Directly examining the evoked potentials from the non-prepulse tests, the N1 CAEP component showed clear and predictable changes according to the strength of the stimulus that induced it. In general, it was broadly observable that increases in stimulus intensity yielded matching increases in the N1 component amplitude. It is worth noting that the 70–80, 80–90, 80–100, and 100–110 dB comparisons were not significantly different from each other at either the Fz or Cz channels, suggesting that similar decibel levels evoke similar responses. Conversely, the remaining decibel level comparisons were found to be significantly different in both the Fz and Cz channels, showing consistency between the two recording sites. These results, in conjunction with the significant main effect, lend credence to the idea that the neural encoding for sound volume is directly linked to the amplitude of the N1 component of cortical auditory evoked potentials. The identical representation and statistics between the two channels would also suggest that future examinations of the CAEP based on scalp EEG do not need to make any major allowances for the recording electrode; both yield acceptable results.

Similar examination, when performed on the prepulse trials, yielded a contrasting similarity across most decibel levels. The 100 dB stimulus showed the greatest amplitude difference, generating CAEP N1 amplitudes that were significantly higher than the 90 dB stimulus in both of the targeted channels. Most stimuli, however, yielded similar CAEP amplitudes in the presence of a prepulse. These results would suggest that prepulse inhibition can, in large part, be objectively observed by examining the auditory evoked potentials. As CAEPs have already been utilized to provide measures of recovery in comatose patients, the ability to similarly measure prepulse inhibition may expand their prognostic capabilities in diseases that present with known PPI alterations. Curiously, the 100 dB stimulus did not show the same level of inhibition as the 110 dB; the 110 dB pulse lead to the highest amplitude N1 component and showed significant differences from low-volume CAEPs, while the addition of a prepulse resulted in an N1 amplitude that was not significantly different from those produced by the 70, 80, 90, or 100 dB stimuli. It would be a logical conclusion that louder stimuli would be more subject to inhibitory effects, though future research would need to explore this in more depth as there is no evident basis for this phenomenon. In contrast to the N1, which showed a significant main effect for stimulus volume, the P2 amplitude showed non-significant main effects. This may indicate that the P2 can serve as a more stable and resilient marker of audition. Theta power then appeared to be a more moderate marker, showing a significant main effect for volume levels in the Cz channel alone.

Direct comparison between the prepulse and non-prepulse stimuli yielded both predictable and unexpected results. When directly compared using FDR-corrected t-tests, measurements of the Fz N1 amplitude showed significant differences in the 70 and 110 dB prepulse conditions, clarifying the significant interaction found by the ANOVA. When observed in prepulse conditions, 110 dB-evoked N1 amplitude was significantly reduced at both the Cz and Fz channels. The P2 component amplitude evoked in response to the 110 dB stimulus also showed significant reductions in both the Fz and Cz channels. While the 70 dB comparison yielded similarly significant results, the introduction of a prepulse yielded contrasting results – the N1 and P2 amplitudes increased in the presence of a prepulse at both the Fz and Cz channels, though only the N1 increases were significant. Considering the significant decibel level changes, prepulse effects, and interactions for the N1 component, this marker may be considered the most variable observed signal. The theta band power also showed results that were similar to those observed in the N1 amplitude comparison – significant decreases at 110 dB with increases in response to the 70 sB stimulus – though with less significant interactions. Theta results, overall, align with the N1 results and are somewhat expected, as literature has suggested that auditory-evoked theta oscillations may be inhibited by prepulses in frontocentral locations^41^.

While the prepulse inhibition observed at 110 dB is a logical and anticipated result, the prepulse-induced facilitation evoked by the 70 dB stimuli is curious and finds itself somewhat at odds with the existing hypotheses of prepulse inhibition. The conventional “interruption” and “protection” hypotheses suggest that a startling acoustic stimulus would interrupt the processing of the prepulse, and that the brain protects this processing, reducing the processing of the startling stimulus. This, however, does not explain the observed the prepulse facilitation (PPF) observed at low volumes: while prepulse facilitation is a recognized phenomenon, previous reports have shown it in cases with minimal intervals between the pulse and prepulse and have not reported volume dependence^29^. The findings presented here, however, suggest that prepulse facilitation can be observed when a prepulse is present before a mild, non-startling pulse. To our knowledge, this finding is the first to indicate that prepulse facilitation can be induced at moderate lead intervals by the modulation of stimulus intensity. The fact that this occurs across all biomarkers, with significant results observed in the N1 and theta power, suggests that this phenomenon is fairly reliable and not the result of errant activity or signals. Even more, the observation of contrasting inhibition and facilitation at different ends of the volume spectrum may suggest that the functional difference between the PPI and PPF phenomena may not be as complete as once believed. Facilitation here was observed at a lead interval that has previously only been use to explore prepulse inhibition. This makes the current finding a necessary point of consideration for future prepulse studies; it cannot be assumed that low-amplitude stimuli will be free from prepulse effects. Such an assumption may introduce unwanted confounds by unintentionally activating the mechanism of PPF, clouding results. Even further, the evocation of both PPI and PPF through the modulation of amplitude alone may indicate that the two phenomenon are not the result of completely separate mechanisms. If PPI and PPF do indeed share some part of the same underlying mechanism, the currently accepted hypotheses would need reconsideration: a unilateral protective influence from moderate-lead prepulses will not account for the observed facilitation.

The study presented here has sought to provide a basis of information regarding CAEPs and PPI/PPF, however there are some limitations to be acknowledged. First, the subject population observed here was fairly young. There is evidence to suggest that CAEP representations change with age^48^, so it may be necessary to expand future study to include multiple age groups. Secondarily, the observed PPF was found primarily in trials where the pulse and prepulse were of equivalent volume. Though it is believed that the lower volume main pulse was the contributing factor for facilitation, it is worth considering that the PPF may occur when the prepulse and main pulse are of similar strength, presenting another potential area for future study. Finally, the current study does not address the P1 CAEP component as it could not be regularly or reliably characterized from the tested subjects. Despite these shortcomings, the results presented here provide potentially valuable information regarding how CAEPs evolve with volume and how PPI functions in contrast, which may in turn enable both future studies and clinical applications.

The auditory startle response is a ubiquitous, well-conserved, and complex reflex that is known to interrupt both physical and cognitive processes. The addition of a prepulse is known to dampen the startle response, though the underlying mechanism for it remains unknown. By examining the N1 CAEP component, a known marker for audition, in an EEG paradigm that presented a variety of stimulus volumes with and without prespulses, distinct patterns and hierarchies were discerned. Direct comparison between conditions with and without prepulses yielded significant differences at both high and low volumes in terms of both N1 amplitude and theta power, with lower amplitudes showing a curious facilitation for both factors. While the present study is by no means a conclusive investigation, the results presented here serve to further the basis of understanding for future tests and may indicate a need to reevaluate or expand the current hypotheses surrounding prepulse inhibition.

## Methods

### Participants

Sixteen healthy subjects(6 female and 10 male, ages 19–27), were recruited to participate in the study under a protocol approved by the University of Houston Institutional Review Board and experiments were performed in accordance with the relevant guidelines and regulations. All participants provided written informed consent and were initially screened to ensure no history of neurological or psychiatric problems, cardiovascular disease, or aural dysfunction. None of the screened subjects met the exclusion criteria and all sixteen recruited subjects were tested in the protocol.

### Materials

All screening and testing was performed on the 2^nd^ floor of the Science and Engineering Research Center on the University of Houston main campus in Houston, Texas. Experiments were performed in an acoustically isolated room with a background noise of ~40 dB. EEG data was recorded using a 64-channel Brainvision ActiCap system (Brain Products, Germany), with the Oz, PO10, and PO9 electrodes sacrificed to measure the sternocleidomastoid muscle, right biceps muscle, and EKG, respectively. The AFz and FCz electrodes were used as the ground and reference electrodes for recording, respectively. The experimental paradigm was designed and run using E-prime 2.0 (Psychology Software Tools, Inc.), with auditory stimuli presented via a JBL Eon speaker (JBL EON 515) placed 12 inches behind the subject. Auditory stimuli consisted of 500 Hz pulses with acoustic intensities of 70, 80, 90, 100, and 110 dB, with a duration of 0.1 s. During trials that featured a prepulse, a 500 Hz, 70 dB prepulse stimulus was presented 50ms prior to the main pulse^4, 20, 34, 49^. EEG data was recorded using BrainVision Recorder (Brain Products, Germany) at a sampling rate of 5000 Hz and a resolution of 0.1μV per bit.

### Experimental Paradigm

**Figure 5:**
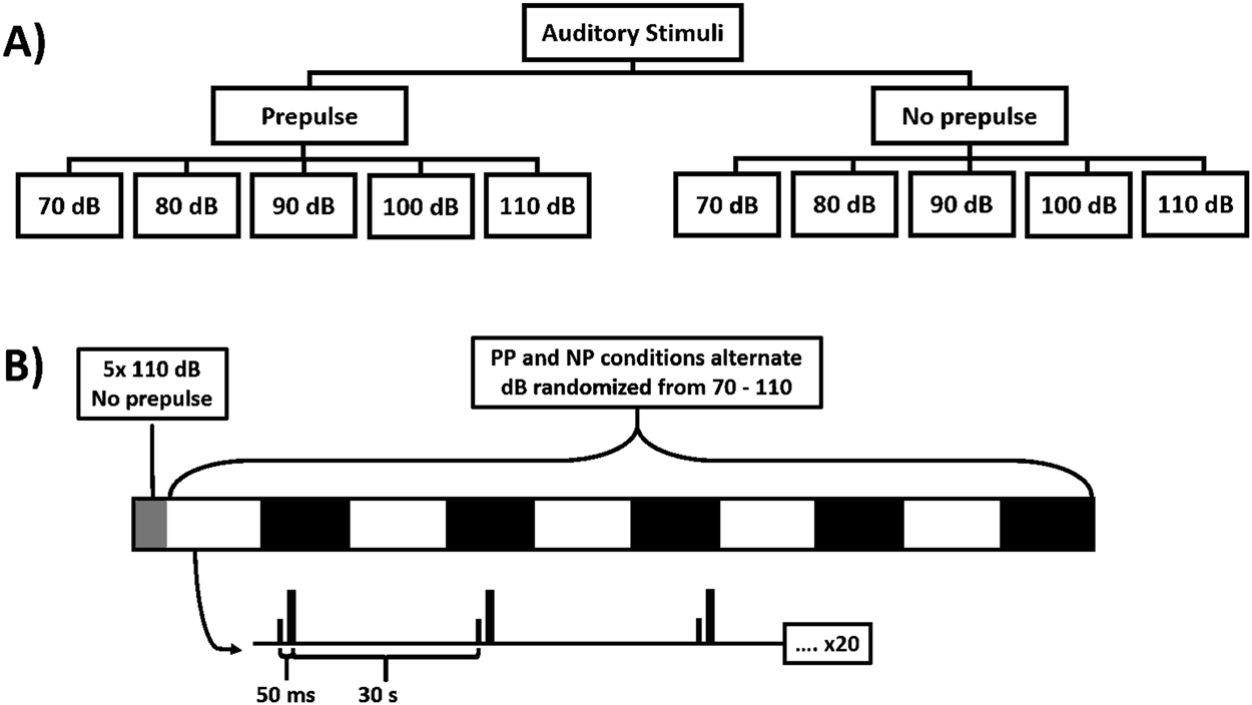
The experimental paradigm. (**A**) shows the 10 individual stimulus conditions, split into 2 conditions (Prepulse (PP) and No prepulse (NP)) and 5 volume levels. (**B**) shows the structure of the paradigm itself, with 10 individual trials consisting of 20 pulses each and preceded by 5 non-prepulse 110 dB pulses. PP and NP conditions were alternated with volume levels randomly presented. Prepulses played 50ms prior to the main pulse and a 30 s interstimulus interval was used.

Subjects were seating upright in a lit room and instructed to remain at rest with their eyes open. The experimental paradigm featured a total of 10 trials split evenly into two conditions: prepulse (PP) and non-prepulse (NP). The 5 trials in each condition were further split into the 5 aforementioned decibel levels (70, 80, 90, 100, and 110 dB) (Fig. 5A). Prepulses were 70 dB, 500 Hz, and 0.1 s in duration, presented 50ms prior to the main pulse. Prior to the main paradigm, 5 non-prepulse, 110 dB stimuli were presented to habituate the subject’s startle reflex and reduce motion artifacts in the subsequent trials. Each trial then consisted of 20 main pulses at the set volume level separated by a 30 s interstimulus interval. Trials alternated in a fixed manner between NP and PP conditions with volume levels randomized without replacement. Figure 5B depicts the overall structure of the paradigm.

### Data Analysis

Data analysis was performed using the Brainvision Analyzer software suite (Brain Products, Germany) and focused on the Fz and Cz channels where CAEPs may be most readily observed^5^. Signals were re-referenced to a common ground and signals were down-sampled to 1000 Hz. Band pass filtration was performed from 1–40 Hz, with a 60 Hz notch filter and a 48 dB/s rolloff. EEG data were referenced to a common average and cardiobalistic artifacts were removed by template subtraction^50^. Independent component analysis was applied to correct ocular artifacts (blinks, saccades) and motion artifacts. One subject was removed from analysis due to poor signal quality, with the fifteen remaining subjects processed fully (n = 15). The resulting data was segmented from 1000ms prior to the main pulse to 1000 ms after and averaged, with baseline correction applied (baseline data was taken from −1000 to −500 ms to avoid overlap with prepulses and anticipation/attentional shifts). The amplitudes of the N1 and P2 components were recorded, along with the averaged theta power. Repeated measures (within subjects) ANOVAs were used with serial two-tailed T-tests were employed as post-hoc tests, with the false discovery rate (FDR) and T1 error rate controlled using the Benjamini-Yekutieli procedure^51^.

### Statistical Methods

Due to the issue of dependency in our experiment and a lack of appropriate post-hoc tests, serial T-tests were adopted with Benjamini-Yekutieli False Discovery Rate analysis to control for type 1 error and increase statistical power. The departments of mathematics at the University of Houston and Houston Baptist University were consulted prior to adopting this method and theorems were review to ensure that the multiple comparison correction would be valid under the assumption of dependency.
